# Comparative Analysis of Exo- and Endonuclease Activities of APE1-like Enzymes

**DOI:** 10.3390/ijms23052869

**Published:** 2022-03-06

**Authors:** Anastasiia T. Davletgildeeva, Alexandra A. Kuznetsova, Darya S. Novopashina, Alexander A. Ishchenko, Murat Saparbaev, Olga S. Fedorova, Nikita A. Kuznetsov

**Affiliations:** 1 Institute of Chemical Biology and Fundamental Medicine, SB RAS, 630090 Novosibirsk, Russia; davleta94@gmail.com (A.T.D.); sandra-k@niboch.nsc.ru (A.A.K.); danov@niboch.nsc.ru (D.S.N.); 2 Groupe Mechanisms of DNA Repair and Carcinogenesis, CNRS UMR9019, Gustave Roussy Cancer Campus, Université Paris-Saclay, CEDEX, F-94805 Villejuif, France; alexander.ishchenko@gustaveroussy.fr (A.A.I.); murat.saparbaev@gustaveroussy.fr (M.S.); 3 Department of Natural Sciences, Novosibirsk State University, 630090 Novosibirsk, Russia

**Keywords:** DNA repair, apurinic/apyrimidinic endonuclease, abasic site, damaged nucleotide, endonuclease activity, 3′-5′ exonuclease activity

## Abstract

Apurinic/apyrimidinic (AP)-endonucleases are multifunctional enzymes that are required for cell viability. AP-endonucleases incise DNA 5′ to an AP-site; can recognize and process some damaged nucleosides; and possess 3′-phosphodiesterase, 3′-phosphatase, and endoribonuclease activities. To elucidate the mechanism of substrate cleavage in detail, we analyzed the effect of mono- and divalent metal ions on the exo- and endonuclease activities of four homologous APE1-like endonucleases (from an insect (Rrp1), amphibian (xAPE1), fish (zAPE1), and from humans (hAPE1)). It was found that the enzymes had similar patterns of dependence on metal ions’ concentrations in terms of AP-endonuclease activity, suggesting that the main biological function (AP-site cleavage) was highly conserved among evolutionarily distant species. The efficiency of the 3′-5′ exonuclease activity was the highest in hAPE1 among these enzymes. In contrast, the endoribonuclease activity of the enzymes could be ranked as hAPE1 ≈ zAPE1 ≤ xAPE1 ≤ Rrp1. Taken together, the results revealed that the tested enzymes differed significantly in their capacity for substrate cleavage, even though the most important catalytic and substrate-binding amino acid residues were conserved. It can be concluded that substrate specificity and cleavage efficiency were controlled by factors external to the catalytic site, e.g., the N-terminal domain of these enzymes.

## 1. Introduction

Apurinic/apyrimidinic (AP)-endonucleases are enzymes essential for abasic-site cleavage in the base excision repair (BER) pathway [1,2,3]. As a result of the AP-endonuclease action, a single-nucleotide gap with 3′-OH and 5′-phosphate is formed [4,5]. Nonetheless, AP-endonucleases can recognize not only AP-sites, but also some damaged nucleotides containing a modified base and catalyze hydrolysis of the phosphodiester bond on the 5′ side of the damaged nucleotide. The activity of AP-endonucleases toward a damaged-base containing DNA was first reported for Nfo from *Escherichia coli* [6,7,8,9], and later described for human APE1 [10,11,12] and other APE1-like enzymes [13]. AP-endonucleases can recognize damaged nucleotides such as 5,6-dihydrouridine, α-anomers of nucleotides, 1,*N*^6^-ethenoadenosine, uracil (U), and other modified residues [10,14]. Moreover, they have 3′-5′ exonuclease [15,16,17] and endoribonuclease [18,19,20,21] activities toward undamaged DNA and RNA, respectively.

Successful characterization of crystal structures of human APE1 [22,23,24,25] bound to DNA has revealed features of enzyme–DNA contacts. A DNA-binding site consists of Arg73, Ala74, Lys78, Tyr128, Arg156, Arg181, Asn222, Asn226, and Thr268. Human APE1 possesses two amino acid residues, Arg177 and Met270, that are inserted into the DNA helix after the eversion of a damaged nucleotide. Amino acid residues Asp70, Glu96, Tyr171, Asp210, Asn212, Asp308, and His309 are responsible for the coordination of cofactor ion Mg^2+^ and for cleaving off the phosphate group of the damaged nucleotide. Despite the successful characterization of crystal structures of human APE1 bound to various metal ions and/or to a substrate or product, the location, stoichiometry, and catalytic function of the divalent metal cation are still being debated [22,26,27,28,29,30,31,32,33,34,35]. For instance, a metal-binding site was identified in the first reported APE1 crystal structure containing one Sm^3+^ ion in the active site, which is often referred to as the “A-site” [26]. Two structures of DNA-free APE1 have been published with Pb^2+^ ions in the active site [28]. Both structures contain a Pb^2+^ ion at the A-site, and one of the structures has the second Pb^2+^ ion, at the “B-site”, coordinated by residues Asp210, Asn212, and His309. A “moving-metal mechanism” has been proposed, where one Mg^2+^ ion moves from the “B-site” to the “A-site” during substrate cleavage [31]. In later studies, the structure of human APE1 has been solved at a 1.92 Å resolution with a single Mg^2+^ ion in the active site [29]. The structure revealed ideal octahedral coordination of Mg^2+^ via two carboxylate groups (Asp70 and Glu96) and four water molecules. The binding site for Mg^2+^ in the new structure was the same as that observed for the surrogate metals Sm^3+^ and Pb^2+^ in previously published structures of DNA-free APE1. The latest structural research on human APE1 showed that Mg^2+^ repositioning was facilitated by the structural plasticity of Glu96 in the active site of the enzyme [34]. Structures of an enzyme–product (EP) complex and enzyme–substrate (ES) complex with a single Mg^2+^ ion in the active site were determined recently [24,25]. In the EP complex, Mg^2+^ is coordinated directly by Glu96, 3′-OH, a nonbridging O atom of the nascent 5′-phosphate, and by three water molecules, one of which is bound to Asp70.

The role of the metal ion and several catalytic mechanisms were recently carefully examined by the molecular mechanics methodology [36]. It was suggested that Mg^2+^-bound water triggers leaving-group departure by neutralizing the 3′-hydroxyl of the neighboring nucleotide. Those authors proposed that the metal ion facilitates the departure of the leaving group through proton transfer from a water molecule. His309, Tyr171, and Asn212 stabilize the increased charge forming on the substrate backbone as the reaction proceeds. Asn212 and Tyr171 also play key roles at the start of the reaction by positioning the Asp210 base and the nucleophilic water molecule in the active site, respectively. Although it has been suggested [37,38] that His309 is neutral and initiates the reaction by activating a water nucleophile, calculations made in [36] indicate that His309 must be protonated to help neutralize the charge on the phosphorane intermediate, in good agreement with NMR and crystallographic data [24,25,39].

In our previous study [35] to determine whether the observed rate enhancements by Mg^2+^ were the result of nonspecific electrostatic effects, we added various concentrations of monovalent cations (K^+^) to reaction mixtures containing 5.0 mM Mg^2+^. It was shown that the initial DNA binding efficiency significantly decreased at a high concentration of monovalent K^+^ ions, indicating the involvement of electrostatic interactions in this stage.

Not so long ago, a mechanism of target nucleotide recognition by human AP-endonuclease APE1 was suggested based on pulsed electron–electron double resonance (PELDOR) spectroscopy [40] and pre-steady-state kinetic analysis of the enzyme’s and DNA’s conformational changes during DNA binding [27,41,42]. The plasticity of the active-site pocket, which allows the pocket to accommodate different damaged nucleotides, has been demonstrated by MD simulations [42]. By summarizing the common strategies of target nucleotide recognition, some authors have hypothesized the mechanism of this process of AP-endonucleases [40,43]. In this mechanism, the nonspecific contacts between amino acid residues of the DNA-binding site and DNA backbone serve to sculpt the DNA structure for the bending of the double helix, local DNA melting, and for damaged-nucleotide eversion.

**Figure 1:**
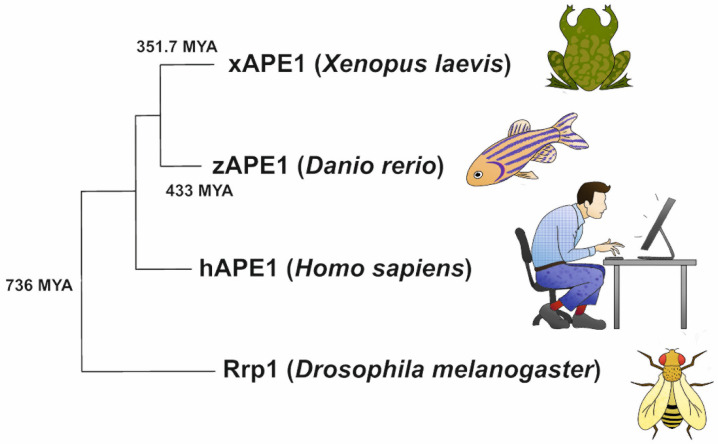
Phylogenetic analysis of the C-catalytic domains of the APE1-like enzymes. Phylogenetic tree was created using the maximum likelihood method (500 replicates) and Jones–Taylor–Thornton (JTT) model by Mega 11 software [44]. Mean divergence times for the species were taken from the TimeTree database and are represented in million years ago (MYA).

Despite the common strategy of target nucleotide recognition, it was recently found [13] that the activity toward DNA containing a damaged base differs among highly homologous APE1-like enzymes. Investigators have revealed significant variation of damaged-DNA cleavage efficacy among such enzymes by comparative kinetic analysis of homologous APE1-like endonucleases—human APE1 (hAPE1), an insect AP-endonuclease (*Drosophila melanogaster* Rrp1), an amphibian AP-endonuclease (*Xenopus laevis* APE1 (xAPE1)), and a fish AP-endonuclease (*Danio rerio* APE1 (zAPE1))—during their interaction with a DNA substrate containing an F-site ((2*R*,3*S*)-2-(hydroxymethyl)-3-hydroxytetrahydrofuran: an analog of an AP-site), 1,*N*^6^-ethenoadenosine, 5,6-dihydrouridine, uridine (U), or the α-anomer of adenosine. Although these AP-endonucleases belong to the evolutionary distinct organisms (Figure 1), their catalytic domains remained largely conserved. An alignment of sequences of the catalytic domain of the four AP-endonucleases [13] revealed that almost all DNA-binding amino acid residues were identical among all these enzymes, except Arg181 (the amino acid numbering corresponds to hAPE1 sequence), which is replaced by Asn in Rrp1. Intercalating (Arg177 and Met270) and catalytic residues also proved to be identical, with a single substitution of Asp70, which coordinated a Mg^2+^ ion, by Ala in the case of Rrp1. It is worth noting that two amino acid residues of the damaged-base-binding pocket (Asn229 and Ala230) were replaced by Thr and Pro in xAPE1. Finally, the researchers proposed [13,42] that the key factor of damage recognition and cleavage efficacy was related to the fine conformational tuning inside the active site. Therefore, the conformational rearrangements inside the active site must be the driving force behind the processes catalyzed by APE1-like enzymes.

To investigate the mechanism of target nucleotide recognition and cleavage by such homologous enzymes in-depth, in the present study, we evaluated the effect of mono- and divalent metal ions on the exo- and endonuclease activities of four APE1-like endonucleases: hAPE1, *D. melanogaster* Rrp1, xAPE1, and zAPE1. Direct detection of the product formation by polyacrylamide gel electrophoresis (PAGE) allowed for estimating the efficiency of the enzymatic cleavage of model substrates. A model DNA substrate containing an abasic site, as well as an undamaged DNA duplex substrate containing a 5′ dangling end and a short hairpin-folded RNA substrate, were used to compare the AP-endonuclease, 3′-5′ exonuclease, and endoribonuclease activities of the four AP-endonucleases at various concentrations of metal ions.

## 2. Results

### 2.1. AP-Endonuclease Activity

**Figure 2:**
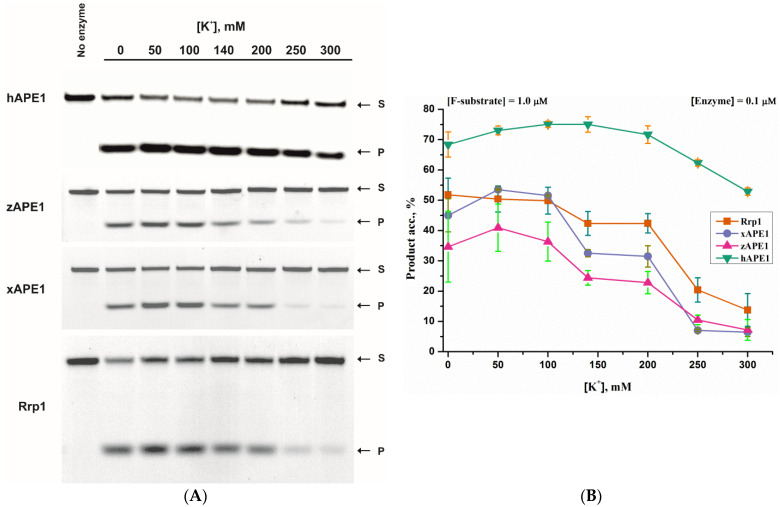
The influence of monovalent cations (K^+^) on the AP-endonuclease activity of the enzymes under study. Accumulation of a reaction product *P* from F-substrate *S* as determined by PAGE (**A**). Dependences of the cleavage efficacy on K^+^ concentration (**B**). The error was calculated by averaging each data point over at least three replicates.

To ascertain the nonspecific electrostatic effect of monovalent cations (K^+^) on the cleavage efficacy of the F-site-containing DNA duplex by APE1-like enzymes, a PAGE assay of the product accumulation was carried out first (Figure 2). The data revealed that the AP-endonuclease activity of all of the tested enzymes had a wide bell-shaped dependence on the K^+^ concentration, in good agreement with a previously reported-steady-state study on DNA binding and catalysis of hAPE1 [35].

In the case of enzymes zAPE1, xAPE1, and Rrp1, their activity was significantly weaker than that of hAPE1. The enzymes were active even in the absence of K^+^ ions and their presence enhanced the reaction; the most efficient cleavage occurred in the presence of 50–100 mM K^+^. The activities of xAPE1 and Rrp1 were approximately equal, and substrate conversion reached ~50%. In the same K^+^ range, zAPE1 cleaved only ~40% of substrate *S* to generate product *P* (Figure 2B). With a further increase in the concentration of K^+^ ions up to 200 mM, the activity toward the F-site-containing substrate did not change noticeably for hAPE1, but visibly decreased for the other three enzymes and could be ranked as Rrp1 > xAPE1 > zAPE1. With an increase of K^+^ concentration to 300 mM, the activities of these three enzymes noticeably were diminished. hAPE1 exhibited an almost unchanged level of F-site cleavage between 50 and 200 mM K^+^, and the activity slightly declined with a further increase of K^+^ concentration.

**Figure 3:**
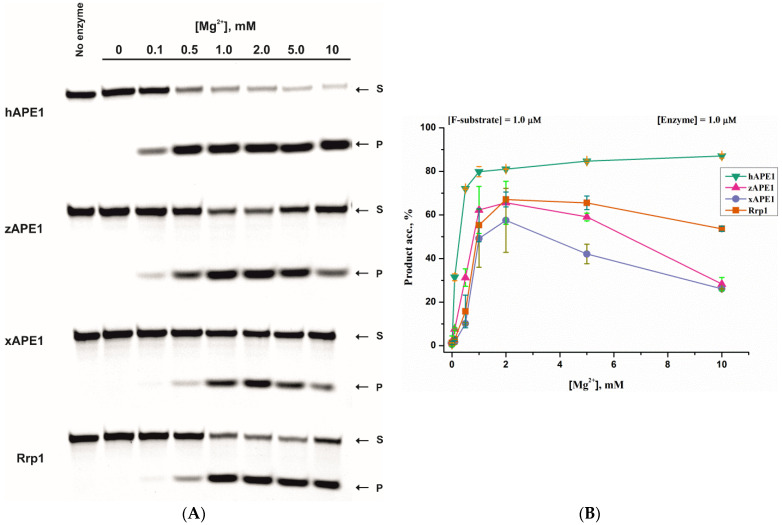
The influence of divalent cations (Mg^2+^) on the AP-endonuclease activity of the four enzymes. Accumulation of reaction product *P* from transformation of F-substrate *S* as determined by PAGE (**A**). Dependences of the cleavage efficacy on the Mg^2+^ concentration (**B**). The error was calculated by averaging each data point over at least three replicates.

The impact of Mg^2+^ concentration on the F-site-containing-DNA cleavage was analyzed by PAGE (Figure 3). For hAPE1, the efficacy of the product accumulation increased up to 1.0 mM Mg^2+^ concentration and was stable within the 1.0–10.0 mM range. In contrast, other APE1-like enzymes manifested bell-shaped dependences with a maximum at 1.0–2.0 mM MgCl_2_. With a further increase of Mg^2+^ concentration, the activity of Rrp1 decreased more slowly than the activity of xAPE1 and zAPE1. There was ~60% substrate conversion to the product in the case of Rrp1 and ~30% for xAPE1 and zAPE1 at 10 mM Mg^2+^. The inhibition of DNA cleavage by a high Mg^2+^ concentration could be explained by the metal binding at the DNA-binding site, resulting in a disturbance of contacts in the active site of the enzymes.

### 2.2. 3′-5′ Exonuclease Activity

**Figure 4:**
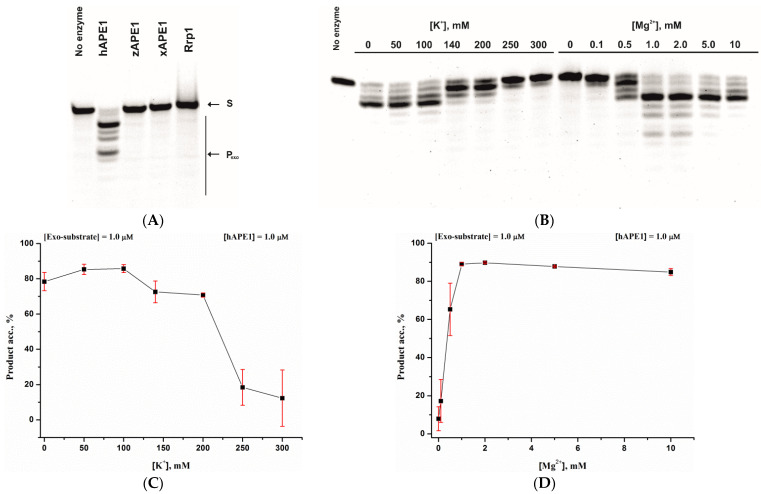
PAGE assays of the 3′-5′ exonuclease activity of the APE1-like enzymes. A comparison of reactions of 3′-5′ exonucleolytic degradation of recessed DNA in presence of 1 mM Mg^2+^ and 50 mM K^+^ (**A**). The impact of K^+^ or Mg^2+^ on the 3′-5′ exonucleolytic DNA cleavage during the interaction with hAPE1 (**B**). Dependences of the cleavage efficacy of hAPE1 on K^+^ (**C**) or Mg^2+^ (**D**) concentration. Error was calculated by averaging each data point over at least three replicates.

Although the activity toward the main biological substrate containing an abasic site was very similar among the APE1-like endonucleases, the capacity for the 3′-5′ exonucleolytic degradation of DNA significantly differed among the homologues enzymes. As shown in Figure 4A, only hAPE1 possessed a pronounced 3′-5′ exonuclease activity. Therefore, the dependences of the 3′-5′ exonucleolytic cleavage of undamaged DNA on MgCl_2_ or KCl concentrations were determined only for hAPE1 (Figure 4B). Of note, the broad bell-shaped K^+^ dependence (Figure 4C) and the maximum of activity at 1.0 mM Mg^2+^ (Figure 4D) were very similar between the AP-endonuclease (Figure 2 and Figure 3) and 3′-5′ exonuclease (Figure 4) activities of hAPE1. It has been shown by Wang Y. et al. that zAPE1 has a very weak 3′-5′ exonuclease activity, which is ~100-fold less than the AP-endonuclease activity of this enzyme [45]. Moreover, to date, there have been no studies on the 3′-5′ exonuclease activity of the xAPE1 enzyme. Overall, the barely noticeable 3′-5′ exonucleolytic degradation of the model DNA by these enzymes was consistent with the literature data and most likely was not due to the plasmid construction procedure used to produce these enzymes in the *E. coli* expression system.

On the other hand, it was unexpectedly found that Rrp1 possessed no significant 3′-5′ exonuclease activity, as demonstrated 30 years earlier [46], when it was first reported that this enzyme could implement 3′-5′ exonucleolytic degradation of DNA. Nevertheless, it was shown later that the 3′-5′ exonuclease activity was dependent on a DNA context [47]. It has also been demonstrated [48] that, unlike hAPE1’s, the Rrp1’s 3′-5′ exonuclease activity depends on the presence of the full-sized N-terminal domain in the enzyme. Considering that Rrp1 possesses the largest N-terminal domain compared to the other tested AP-endonucleases, it could be assumed that the structural features of this noncatalytic domain in the full-sized enzyme produced in the *E. coli* expression system could affect the 3′-5′ exonuclease activity of Rrp1.

### 2.3. Endoribonuclease Activity

The key difference in the catalytic mechanisms of phosphodiester bond hydrolysis between RNA and DNA substrates is that the reaction yields different products: 3′-PO_4_^2−^ for RNA and 3′-OH for DNA [49]. Moreover, hAPE1 does not need divalent metal ions to exert its endoribonuclease action [49,50], supporting the above statement about the difference in the catalytic mechanism between DNA cleavage and RNA cleavage. Undamaged RNA fragments are preferentially cleaved at the phosphodiester bond within dinucleotides UA, UG, and CA in single-stranded sequences or weakly paired RNA regions [50]. As revealed recently [51,52,53], a short hairpin folded RNA substrate is cleaved most effectively among various native RNAs tested in an endoribonucleolytic cleavage assay involving hAPE1. Of note, its ability to hydrolyze RNA has been documented with respect to microRNA, c-Myc mRNA, CD44 mRNA, and RNA components of the SARS coronavirus [20,54]. It has been suggested [20] that endoribonucleolytic hydrolysis of mRNA may be one of the major functions of hAPE1 in the cytoplasm.

**Figure 5:**
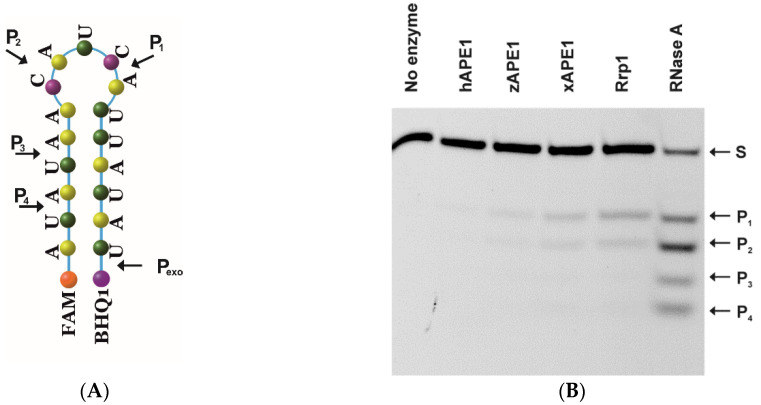

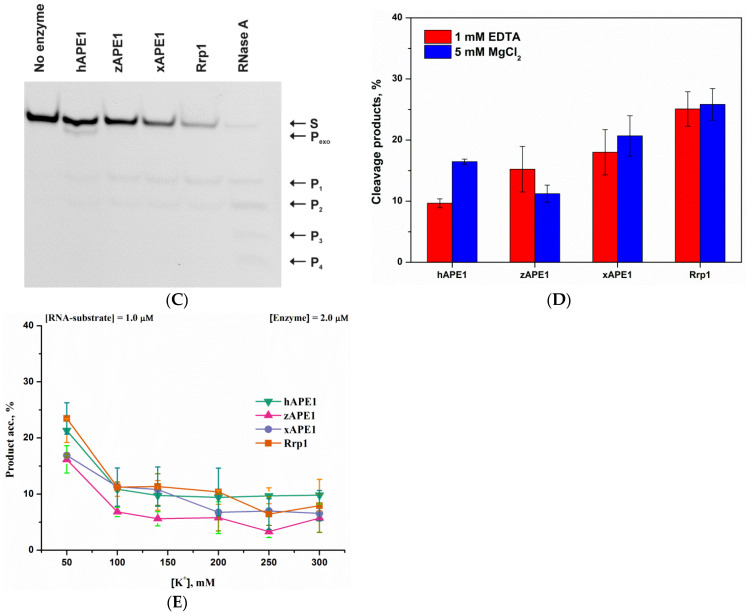
The efficiency of cleavage of the RNA substrate by the four APE1-like enzymes. Positions of the hydrolyzed nucleotides are pointed out by arrows as revealed by RNase A cleavage (**A**). PAGE analysis of the reaction products in the presence of 1.0 mM EDTA (**B**) or 5.0 mM MgCl_2_ (**C**). A comparison of the efficacy rates of cleavage of the RNA substrate by the four APE1-like enzymes (**D**). Dependences of the RNA cleavage efficacy on K^+^ concentration (**E**). Error was calculated by averaging each data point over at least three replicates. Enzyme = 2.0 μM, RNA = 1.0 μM, and reaction time = 1 h.

Therefore, a comparative analysis of the endoribonucleolytic hydrolysis of the model RNA substrate (Figure 5A) by the four APE1-like enzymes was performed next. The interaction of the APE1-like enzymes with the model RNA substrate was evaluated both in the absence of divalent metal ions (Figure 5B) and in the presence of Mg^2+^ (Figure 5C). As revealed by the PAGE assay, four sites of endoribonucleolytic cleavage are presented in the model RNA substrates, which are indicated by arrows in Figure 5A. It was previously found [21] that 3′-5′ endonuclease degradation of RNA could appear only in the presence of cofactor Mg^2+^ ions. Notably, only during the interaction of hAPE1 with the RNA substrate in a buffer supplemented with 5 mM MgCl_2_, was the additional 3′-5′ exonuclease product P_exo_ detected (Figure 5C), implying that the 3′-5′ exonuclease activity of hAPE1 toward the RNA substrate was the most efficient among all of the tested enzymes.

The relative activities of the APE1-like enzymes toward the RNA substrate in the presence of EDTA and Mg^2+^ were determined too (Figure 5D). These data clearly indicated that the endoribonuclease activity of these enzymes slightly increased in the order hAPE1 ≈ zAPE1 ≤ xAPE1 ≤ Rrp1. On the other hand, the dependences of the RNA cleavage efficacy on the concentration of K^+^ were similar among all of the tested enzymes and significantly differed from the dependences of the AP-endonuclease and 3′-5′ exonuclease activities on K^+^ concentration. Indeed, an increase of KCl concentration over 100 mM reduced the RNA cleavage efficiency to the lowest level, ~10%, whereas in the case of DNA cleavage efficiency, this level was reached at 250 mM KCl (Figure 5E), supporting that K^+^ ions efficiently blocked nonspecific electrostatic interactions between the amino acid residues of the active site and RNA substrate backbone.

## 3. Discussion

Despite significant achievements in the understanding of the functional properties of AP-endonucleases, the question of how a given enzyme controls its activity toward various substrates remains unanswered. It has been commonly thought that the main biological function of AP-endonucleases is the hydrolytic cleavage of a DNA strand on the 5′ side of an AP-site and the subsequent formation of a single-strand break with a 5′-deoxyribophosphate and a 3′-hydroxyl group [5,55]. Indeed, our current data on the metal ion dependence of the AP-endonuclease activity revealed a wide bell-shaped dependence of the AP-endonuclease activity on the KCl concentration, as well as strong activation of the four enzymes by 1.0 mM MgCl_2_. These very similar behaviors of all the tested enzymes suggest that the main biological function of AP-endonucleases is highly conserved among evolutionarily distant species.

AP-endonucleases recognize undamaged nucleotides when catalyzing the 3′-5′ exonucleolytic [39,56] or endoribonucleolytic [49] cleavage of DNA and RNA, respectively. Our findings mean that the efficiency of the 3′-5′ exonuclease activity is significantly higher in hAPE1 than in the other three tested enzymes. Moreover, the effect of metal ions on the 3′-5′ exonuclease activity of hAPE1 was very similar to the effect on its AP-endonuclease activity. These data suggest that the 3′-5′ exonuclease activity of hAPE1 performed an important biological function in mammals. Moreover, it should be noted that mammalian cells possess two APE1-like enzymes (APE1 and APE2), and between them, active-site amino acid residues are fully conserved [18,22,57]. Human enzyme hAPE1 has a highly efficient AP-endonuclease activity, and its 3′-end cleansing including 3′-5′ exonuclease activity is substantially weaker. In contrast to hAPE1, the 3′-5′ exonuclease activity of hAPE2 is higher than its AP-endonuclease activity [58]. Furthermore, several other APE1-like AP-endonucleases, such as XthA from *Mycobacterium tuberculosis*, NApe and NExo from *Neisseria meningitides*, and APE1L from *Arabidopsis thaliana,* completely lack the ability to process base-damaged nucleotides despite sharing APE1’s architecture of the catalytic site [59,60,61]. These observations imply that the efficiency of the 3′-5′ exonuclease activity is not controlled by a network of conserved catalytic-active-site amino acid residues, but rather depends on some additional factors, probably such as significantly different N-terminal domains of APE1-like enzymes or the global plasticity of the active-site pocket. This conclusion is in agreement with existing data [13] from pre-steady-state analysis of the interaction of the tested APE1-like enzymes with DNA substrates containing various base-damaged nucleotides. The differences in the rates of DNA substrates’ binding do not translate into significant differences in the efficiency of cleavage of DNA containing a damaged base. Therefore, it is reasonable to conclude that the cleavage efficacy is related to fine conformational tuning inside the active site.

On the other hand, the N-terminal domain differs significantly in size and composition among the tested APE1-like enzymes. They contain a large number of positively charged amino acids (arginines and lysines). Some reports [10,11,62] have revealed that a loss of the N-terminal domain in hAPE1 affects both the rate of formation and the stability of the initial complex in the cases of base-damaged-DNA cleavage and 3′-5′ exonuclease activity. Moreover, investigation of N-terminally truncated hAPE1 mutants has shown that its positively charged lysine-rich region can act as a stabilizer of the binding to nucleic acids [63,64]. In contrast, this domain does not influence the main function of hAPE1 (AP-endonucleolytic incision) [11,65,66] in the context of abasic-DNA processing.

It was found here that all the tested APE1-like enzymes possessed an endoribonuclease activity in the absence of cofactor Mg^2+^, as previously reported for hAPE1 [53], thus pointing to a shared mechanism of RNA cleavage. Our data indicate that endoribonuclease activity of the enzymes increased in the order hAPE1 ≈ zAPE1 ≤ xAPE1 ≤ Rrp1, with a strong inhibition by 100 mM KCl. These results imply a possible important role of the N-terminal domain of these enzymes in the interaction with an RNA substrate.

Overall, in this study, we compared the effects of mono- and divalent metal ions on three types of activities of APE1-like enzymes: AP-endonuclease, 3′-5′ exonuclease, and endoribonuclease abilities. For this purpose, the efficiency of cleavage of model damaged and undamaged DNA and RNA substrates was assessed. It turned out that despite conserved catalytic amino acid residues in the active site and very similar patterns of the main (AP-endonuclease) activity, these enzymes possess significantly different capacities for the 3′-5′ exonucleolytic degradation of undamaged DNA. Among these enzymes, hAPE1 manifested the most efficient 3′-5′ exonucleolytic degradation of the model undamaged DNA, pointing to the biological importance of this function in mammals. On the other hand, our results indicate that variation of the endoribonuclease activity among these enzymes could be attributed to the action of the N-terminal domain, which could bind nucleic acids through electrostatic interactions. The interactions with the N-terminal domain in the context of RNA binding were confirmed by the much higher sensitivity to monovalent ions (K^+^) observed in comparison with other the enzymatic activities.

## 4. Materials and Methods

### 4.1. Cloning of AP-Endonucleases

Synthetic codon-optimized genes of zAPE1 from *D. rerio*, xAPE1 from *X. laevis*, and Rrp1 from *D. melanogaster* were employed to construct expression plasmids. The genes of these enzymes were digested with BamHI and NdeI/NheI restriction enzymes, separated on 1.0% agarose/TAE gels, excised, and purified with the QIAquick PCR Purification Kit (Qiagen, Hilden, Germany). Each gene was cloned into the pET28c expression vector digested by the same restriction enzymes. Ligation products were electroporated into ElectroMAX™ DH10B Competent Cells (Invitrogen, Waltham, MA, USA), purified by means of the Plasmid Miniprep Kit (Merck, Darmstadt, Germany), and sequenced. The obtained constructs expressed the zAPE1, xAPE1, or Rrp1 protein with an N-terminal His-tag.

### 4.2. Enzyme Purification

**Figure 6:**
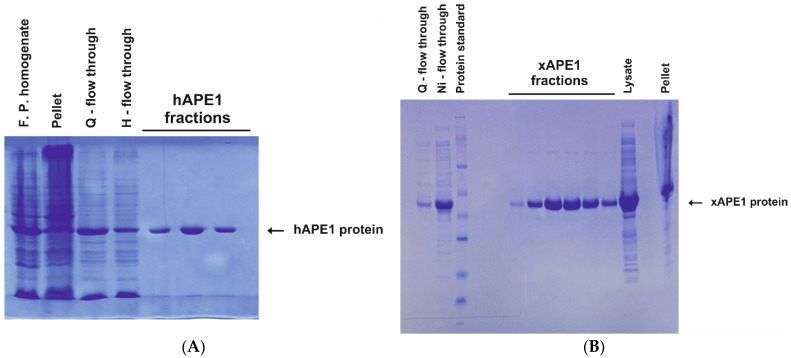
Analysis of the protein purity of the hAPE1 (**A**) and the xAPE1 (**B**) proteins by PAGE. The probes containing the homogenate obtained by the French-Press procedure, the cell pellet, the flow-through fractions after the Q-Sepharose chromatography, and the Heparin (hAPE1) or Ni-chelating (xAPE1) chromatography, as well as the protein fractions obtained by gradient elution of protein from the heparin/Ni-chelating columns were analyzed. Coomassie Brilliant Blue R-250 (Sangon Biotech, Shanghai, China) staining was performed for protein visualization.

hAPE1 was expressed and purified in its native form without tags or other modifications, as described previously [41,67]. Briefly, 1 L of *E. coli* strain Rosetta II (DE3) culture (Invitrogen, Villebon-Sur-Yvette, France) (in Luria-Bertani (LB) broth) carrying the pET11a-hAPE1 construct was grown at 50 μg/mL ampicillin and 37 °C until absorbance at 600 nm (A_600_) reached 0.6–0.7. The enzyme expression was induced overnight at 20 °C with 0.2 mM isopro-pyl-β-D-thiogalactopyranoside (IPTG). The cells were then harvested by centrifugation and re-suspended in 30 mL of buffer I (20 mM HEPES-NaOH pH 7.8, 40 mM NaCl, and 0.1% of NP40) containing a protease inhibitor cocktail (Complete, Darmstadt, Germany). The cells were lysed using a Thermo French Pressure Cell Press. The homogenate was centrifuged at 30,000 rpm for 40 min, and next the supernatant was passed through a column packed with 25 mL of Q-Sepharose Fast Flow (Cytiva, GE Healthcare Life Sciences, Marlborough, MA, USA) and washed with buffer I (20 mM HEPES-NaOH, pH 7.8) containing 40 mM NaCl. The flow-through fractions containing the enzyme were pooled and loaded onto column II (HiTrap-Heparin™, Amersham Biosciences, Uppsala, Sweden). Chromatography was performed in buffer I with a linear gradient of 40→600 mM of NaCl; the solution’s absorbance was detected at 280 nm. All purification procedures were carried out at 4 °C. The purity of the enzyme was determined by gel electrophoresis (Figure 6A). Fractions containing the hAPE1 protein were dialyzed in buffer II (20 mM HEPES-NaOH, 1 mM EDTA, 1 mM dithiothreitol, 250 mM NaCl, 50% glycerol, pH 7.5) and stored at −20 °C.

For the expression of the recombinant enzymes zAPE1, xAPE1, and Rrp1, 1 L of *E. coli* strain Rosetta II(DE3) culture (Invitrogen, Villebon-Sur-Yvette, France) (in Luria-Bertani (LB) broth) carrying the pET28c-APE1 construct was grown at 50 μg/mL kanamycin and 37 °C until absorbance at 600 nm (A_600_) reached 0.6–0.7. The enzyme expression was induced overnight at room temperature with 0.3 mM isopropyl-β-D-thiogalactopyranoside. The cells were then harvested by centrifugation and resuspended in 30 mL of buffer I (20 mM HEPES-KOH pH 7.8, 40 mM NaCl, and 0.1% of NP40) containing a protease inhibitor cocktail (Complete, Darmstadt, Germany). The cells were lysed using a Thermo French Pressure Cell Press. The homogenate was centrifuged at 40,000× *g* for 45 min, and, next, the NaCl concentration in the supernatant was brought to 250 mM (400 mM in case of Rrp1), and the supernatant was passed through a column packed with 25 mL of Q-Sepharose Fast Flow (Cytiva, GE Healthcare Life Sciences, Marlborough, MA, USA) pre-equilibrated in the same buffer. The flow-through fractions containing the enzyme were pooled and loaded onto a 1 mL HiTrap Chelating HP™ column (Cytiva GE Healthcare Life Sciences, Marlborough, MA, USA) charged with Ni^2+^ in buffer III (20 mM HEPES-NaOH pH 7.8, 500 mM NaCl, and 20 mM imidazole). Bound proteins were eluted in a linear 20–500 mM gradient of imidazole. The purity of enzymes was determined by gel electrophoresis, and an example PAGE analysis of purification fractions of xAPE1 is shown in Figure 6B. All purification procedures were carried out at 4 °C. The protein concentration was measured by the Bradford method; the stock solution was stored at −20 °C in 50% glycerol.

The purified proteins containing the His-tag, namely zAPE1, xAPE1, and Rrp1, were subjected to the buffer exchange procedure using the Amicon^®^ Ultra Centrifugal Filters (EMD Millipore, Burlington, MA, USA) before performing the enzyme assays. The resulting buffer contained 50 mM Tris-HCl (pH 7.5), 50 mM NaCl, 1 mM dithiothreitol, and 50% glycerol.

### 4.3. Oligodeoxynucleotides (ODNs)

The synthesis of the oligonucleotides (Table 1) was carried out on an ASM-800 DNA/RNA synthesizer (Biosset, Novosibirsk, Russia) using standard commercial phosphoramidites and CPG solid support from Glen Research (Sterling, VA, USA). The oligonucleotides were deprotected according to the manufacturer’s protocols and purified by high-performance liquid chromatography. Oligonucleotide homogeneity was checked by 20% denaturing PAGE. The concentrations of oligonucleotides were calculated from their A_260_. ODN duplexes were prepared by annealing oligonucleotide strands at a 1:1 molar ratio.

### 4.4. PAGE Experiments

Single-turnover enzyme assays were performed in different reaction buffers consisting of 50 mM Tris-HCl pH 7.5, 0–300 mM KCl, 0.0–10.0 mM MgCl_2_, 1.0 mM dithiothreitol, 0.0–1.0 mM EDTA, and 7% of glycerol (*v*/*v*). AP-endonuclease assays with each substrate were conducted at 25 °C in a 10 µL reaction mixture. Because AP-endonuclease and endoribonuclease activities have different characteristic times of substrate cleavage, the reaction mixtures were incubated for different periods, and the reaction was carried out at different concentrations for the reactants (Table 2). The reaction was initiated by the addition of the enzyme and was quenched with 10 µL of a gel-loading dye containing 7 M urea and 25 mM EDTA, followed by heating at 95 °C for 3 min and loading on a 20% (*w*/*v*) polyacrylamide/7 M urea gel. The hydrolysis of the model RNA substrate by RNase A was performed to visualize cleavage products in a pyrimidine-purine sequence context. The RNase A reaction mixture (20 μL) composed of 3.0 μM substrate and 3.0 nM RNase A in a buffer (50 mM Tris-HCl (pH 8.5), 50 mM NaCl, 1.0 mM EDTA, 1.0 mM DTT, and 9% of glycerol) was incubated at 25 °C for 5 min, quenched with 20 μL of a gel loading solution (7 M urea and 25 mM EDTA), and incubated at 96 °C for 5 min to stop the reaction.

PAGE was performed under denaturing conditions (7 M urea) at 55 °C and a voltage of 200–300 V. The gels were visualized using an E-Box CX.5 TS gel-documenting system (Vilber Lourman, France), and the bands were quantified in the Gel-Pro Analyzer software (Media Cybernetics, Rockville, MD, USA).

### 4.5. Phylogenetic Analysis

The amino acid sequences of the C-terminal catalytic domains of the four tested APE1-like enzymes were obtained from the UniProt database and transferred to Mega 11 software [44]. Using the aligned amino acid sequences, a phylogenetic tree was constructed with Mega 11 software (Figure 1). At this stage, the maximum likelihood (ML) method and Jones–Taylor–Thornton model with 500 bootstrap replicates were used [68]. To identify the pairwise mean divergence times for the species, the TimeTree web resource was used [69]. The graph was drawn according to the phylogenetics generated by the ML method [70].

## Figures and Tables

**Table 1:** DNA substrates used in this study ^a^.

F-substrate	5′–FAM–GCTCAFGTACAGAGCTG–3′ 3′–CGAGTGCATGTCTCGAC–BHQ1–5′
Exo-substrate	5′–FAM–CAGCTCTGTACGTGAGC–3′ 3′–GTCGAGACATGCACTCGTCACCACTGTG–5′
RNA substrate	5′–FAM–r(AUAUAACAUCAUUAUAU)–BHQ1–3′

^a^ FAM is 6-carboxyfluorescein, BHQ1 is black hole quencher, and F is (2R,3S)-2-(hydroxymethyl)-3-hydroxytetrahydrofuran (an abasic-site analog).

**Table 2:** Reaction conditions for the comparison of cleavage activity among APE1-like enzymes.

Substrate	Reactant Concentrations	Reaction Conditions
F-substrate	[enzyme] = 1.0 μM, [substrate] = 1.0 μM	(1) [MgCl_2_] = 5.0 mM, [KCl] = 0–300 mM, 25 °C, 20 s (2) [MgCl_2_] = 0–10 mM, [KCl] = 50 mM, 25 °C, 20 s
Exo-substrate	[enzyme] = 1.0 μM, [substrate] = 1.0 μM	(1) [MgCl_2_] = 5.0 mM, [KCl] = 50 mM, 25 °C, 30 min (2) [MgCl_2_] = 5.0 mM, [KCl] = 0–300 mM, 25 °C, 30 min (3) [MgCl_2_] = 0–10 mM, [KCl] = 50 mM, 25 °C, 30 min
RNA substrate	[enzyme] = 2.0 μM, [substrate] = 1.0 μM	[MgCl_2_] = 0.0 or 5.0 mM, [KCl] = 50 mM, 25 °C, 1 h

## Data Availability

Experimental data are available upon request to N.A.K. Tel. +7-383-363-5174, E-mail: nikita.kuznetsov@niboch.nsc.ru.
